# Analysis of Air Pollution Based on the Measurement Results from a Mobile Laboratory for the Measurement of Air Pollution

**DOI:** 10.3390/ijerph192013474

**Published:** 2022-10-18

**Authors:** Elwira Zajusz-Zubek, Zygmunt Korban

**Affiliations:** 1 Department of Air Protection, Faculty of Energy and Environmental Engineering, Silesian University of Technology, 44-100 Gliwice, Poland; 2 Department of Safety Engineering, Faculty of Mining, Safety Engineering and Industrial Automation, Silesian University of Technology, 44-100 Gliwice, Poland

**Keywords:** environmental monitoring, Czekanowski’s method, linear ordering of the “objects”

## Abstract

One of the most important effects of the smog phenomenon is the presence of high concentrations of substances hazardous to human life and health in the air. Environmental monitoring, including the monitoring of substances hazardous to human life or health, is an element of preventive measures that allow to identify current hazards and to define future actions aimed to improve (protect) the state of the environment. The article presents the results of measurements of the concentration of PM_10_ and PM_2.5_ as well as SO_2_, NO, NO_x_ and O_3_ based on a mobile laboratory located on the campus of the Silesian University of Technology. By treating the following weeks as “objects”, points in the multidimensional space (the concentrations of PM_10_ and PM_2.5_ as well as SO_2_, NO, NO_x_ and O_3_ were the measures/describing features), similarities between them were determined, and then they were grouped into the “summer period” (from 01/04/2020 to 30/09/2020) and “winter period” (from 01/01/2020 to 31/03/2020 and from 01/10/2020 to 31/12/2020). The article aimed to determine a linear ordering of weeks divided into the “summer period” and the “winter period”. The software MaCzek v. 3.0 (an application working in Windows) was used in the computing layer.

## 1. Introduction

Numerous toxicological and epidemiological studies provide more and more evidence that air pollution is related to health problems [1,2]. The exposure to air pollutants is highly dependent on their concentration. Importantly, according to the report by the World Health Organization [3], nine out of ten people breathe polluted air and this causes around seven million deaths worldwide each year. With high levels of particulate matter PM_10_ or PM_2.5_ concentrations, and with gaseous pollutants such as CO, O_3_ and NO_2_ in cities around the world, the weather and specific climatic conditions have a significant impact on the confirmed rise in COVID-19 rates and deaths [4,5].

Most research is currently focusing on the respirable PM_2.5_ fraction, which is considered the most harmful because, when inhaled, fine particles can penetrate deep into the lungs together with organic and inorganic compounds adsorbed on their surface [6]. The concentrations of PM_2.5_ in the air vary significantly in different regions of the world.

Poland is one of the countries where air quality standards have been surpassed in the prevailing area, and according to the European Environment Agency, Poland has for many years been occupying one of the top places in the classification of countries with the most polluted air in Europe. The European Union has implemented a number of legal instruments to change the quality of atmospheric air, and consequently the situation has improved in terms of the content of such pollutants, e.g., lead, sulfur dioxide or benzene. Unfortunately, the fractions of suspended particulate matter PM_10_ and PM_2.5_ and their components, such as benzo(a)pyrene, as well as gaseous pollutants, such as nitrogen oxides (NO_x_), are still a problem in a large area of Poland, where the recorded concentrations are ranked as the highest in Europe. The problem, which negatively contributes to bad air quality in Poland, is the use of old motor vehicles, especially those with diesel engines, which are significant sources of PM emissions. Exceeding the air quality standards for PM_10_ and PM_2.5_ is not only effected by their emission, but also by their gaseous precursors commonly emitted into the atmosphere (mainly SO_2_, NO_x_, ammonia and hydrocarbons). Legal changes involving emissions standards will bring about a further reduction of emissions, mainly in relation to gaseous pollutants, especially sulfur dioxide, nitrogen oxides and PM pollutants. The current state of air quality in Poland, and in many European Union countries, is mainly effected by the so-called low emissions from the household and municipal sector, comprising individual heat generation sources and small municipal heating plants or small service providers, as well as emissions from car transport [7]. The dominant emission sources are energy production and home heating. The factors that negatively affect air quality, especially in the case of low emission sources, are unfavorable meteorological conditions such as: no wind conditions, low temperature or fog. High wind speeds and intense rainfall result in a significant decrease in the concentration of pollutants. There are many reasons for poor air quality in Poland. One of the main ones is the dominance of coal. Despite a significant development of heating systems based on renewable energy sources, taking into account the size of hard coal or lignite resources and energy security, it can be assumed that coal will continue to be the basic fuel in the industrial and communal energy sector and in households. The combustion processes of fuels generate 55 to 80% of the total PM emissions. Coal power plants are a significant source of emissions of most hazardous substances, including bioavailable forms of elements such as: As, Cd, Cr and Ni [8].

This problem does not only concern Poland, but also other countries where energy production is based on coal. Problems related to air quality in the vicinity of large emission sources related to coal processing will be increasing.

According to antismog resolutions in Poland, the combustion of lignite and solid fuels produced with its use, coal sludge, flotoconcentrates and their mixtures, and solid biomass, the humidity of which exceeds 20%, is prohibited.

Wood and coal burning in homes are estimated to cause almost 40% of outdoor fine particulate air pollution.

In this article, measurements of PM10 and PM_2.5_, as well as SO_2_, NO, NO_x_ and O_3_ dust content made on the basis of a mobile laboratory located on the campus of the Silesian University of Technology (Gliwice, 20B Konarskiego St., 50.292934 N, 18.682164 E), were used to develop comparative statements in the adopted measurement periods (weeks). These statements are based on replacing the entire set of characteristics describing the object (primary data) with one variable that is an aggregate (synthetic) quantity. Treating measurement periods as points in a multidimensional space, the relationship for determining Euclidean distances between them and the Czekanowski method for determining clusters of the above-mentioned objects was used. The application of Czekanowski’s method allowed the linear ordering of objects and to determine grouping of the objects.

## 2. Materials and Methods

### 2.1. The Use of Czekanowski’s Procedure in the Process of Data Grouping—Theoretical Basis

In the process of a broadly understood assessment, synthetic measures are becoming more and more important. They make it possible to replace the entire set of features describing the object (primary data, partial assessments) with one variable being an aggregated (synthetic) value. Czekanowski’s procedure (Czekanowski’s diagram, Czekanowski’s diagraphic method), which is the oldest numerical taxonomic method, originally developed for the needs of anthropology [9,10,11], belongs to the category of data grouping/clustering analysis. This method is used especially when complex dependencies between objects in the series prevent an effective use of hierarchical grouping methods. In this method, the starting point is a symmetrical, square Euclid’s distance matrix D between the study objects [9,10,11]:

(1) $$D=\left|d_{\mathrm{rs}}\right|,\;\mathrm{for}\;r,\;s=1,\;2,\;\ldots ,\;n,$$

where:
d_rs_—distance between the r-th and s-th object;
n—number of objects.

The algorithm of proceeding in Czekanowski’s method consists in rearranging the lines and the corresponding to them columns in the matrix D in such a way that the smallest possible elements are along the diagonal of the matrix and that, with the distance from the diagonal, the values of the distance measures become larger and larger. All distance measures are divided into several classes, and then individual classes are assigned graphic symbols, which allows for a visual assessment of fit of the matrix D. In Czekanowski’s method, the matching degree depends on many subjective elements, including, e.g., the adopted average difference between the examined objects (so as to consider it significant) or whether an object that shows close relations with two groups is accepted. The requirement of the maximum concentration of the objects along the main diagonal is possible only by the method of successive approximations, yet it is never known whether the obtained ultimate image is really final [12].

As the final effect of the application of Czekanowski’s method, we obtain [9,10] the so-called ordered distance matrix between objects (linear ordering of the classified objects), which in turn enables to define the so called clusters of objects (objects located close to each other in the multidimensional space).

**Figure 1:**
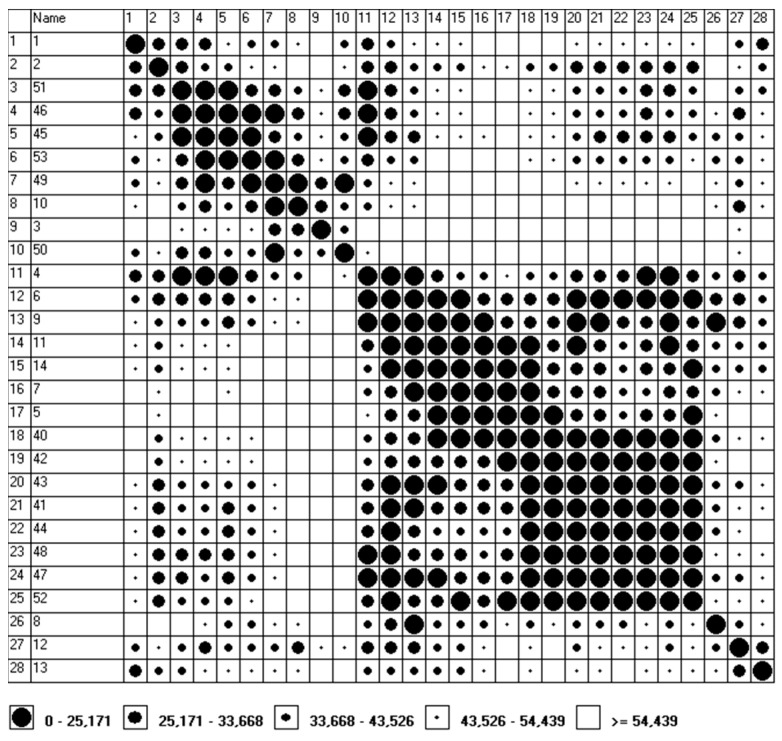
Czekanowski’s diagram—“winter period”. (The MaCzek v. 3.0 program generates the graphic image of the grouping of objects by itself: the first column and the horizontal axis (the row at the top of the drawing) contain the numbers assigned to the following weeks (the second column is the number of the weeks counted from the beginning of the year).)

**Figure 2:**
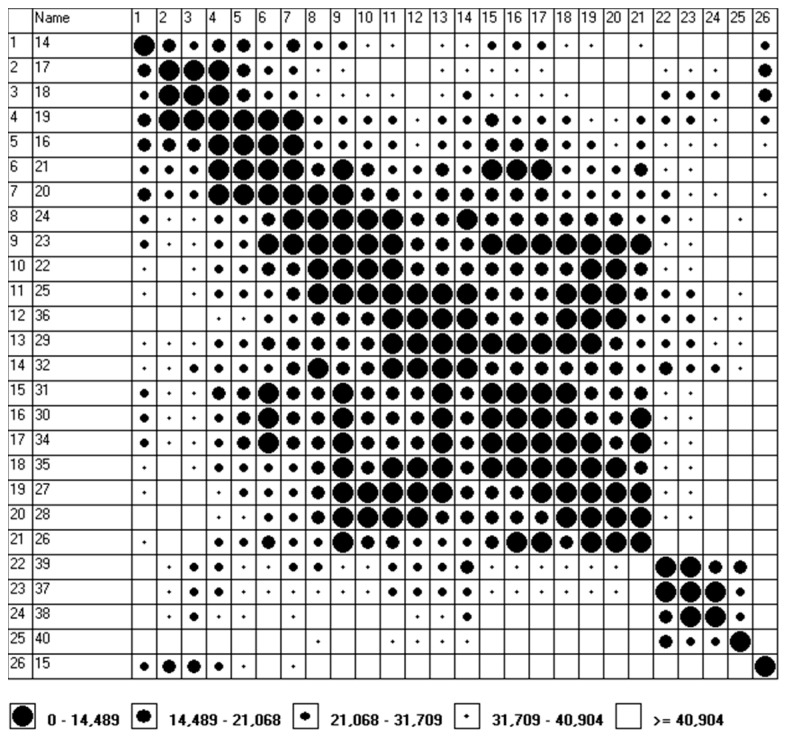
Czekanowski’s diagram—“summer period”. (The MaCzek v. 3.0 program generates the graphic image of the grouping of objects by itself: the first column and the horizontal axis (the row at the top of the drawing) contain the numbers assigned to the following weeks (the second column is the number of the weeks counted from the beginning of the year).)

In the opinion of the Authors, the results of the measurements obtained by stationary environmental monitoring stations supplemented with additional measurements (e.g., the ones carried out on the basis of mobile laboratories), along with selected methods of multicriteria assessment (e.g., Czekanowski’s diagram, Hellwig’s method, CLUSTER procedure under Matlab, etc.) may be used in the so-called environmental management. The authors of the manuscript consider this by creating groups (clusters) of objects characterized by a high degree of mutual similarity, which may be helpful in creating risk maps related to the occurrence of atmospheric pollution. The development of databases containing, for example, information on the emission levels of substances hazardous to human life or health, information on weather conditions and on the location of measuring points will allow to identify areas containing objects similar to each other in terms of the presence of many features occurring at the same time. In 2020, due to the threat posed by the SARS-CoV-2 virus (lockdown), the measurements were carried out only at one measurement point (Gliwice, Konarskiego 20B, 50.292934 N, 18.682164 E), which resulted in the adoption of successive weeks of the year 2020 as “objects”. In addition, the adoption of weeks as “objects” was intended to ensure the appropriate quality of the presented diagrams (Figure 1 and Figure 2), and it was also connected with the limitations of the software used. Program MaCzek v. 3.0 is a Windows-based application (Windows 95 and newer versions) which, in addition to creating Czekanowski’s diagrams, allows also to standardize data and to determine the distances between objects. The maximum size of the diagram covers 250 objects and 100 features describing the object [13]. Ultimately, these “objects” are to be the measuring points located in the area of the Silesian Voivodeship, including places located far away from the existing environmental monitoring stations, in the immediate vicinity of kindergartens, schools, hospitals, etc.

### 2.2. Location of the Measuring Point

Since the fourth quarter of 2018, the Silesian University of Technology has been equipped with a mobile laboratory built on a Ford Transit chassis. The mobile air pollution laboratory is equipped with: SO_2_—T100/Teledyne API analyzer; NO_x_—T200/Teledyne API analyzer; O_3_—T400/Teledyne API analyzer; PM_10_/PM_2.5_ BAM1020 m; a meteo kit WS 500 Lufft, an Envimet Services intake; an Envimet Services calibration system; an Envimet Services data logger with a display; an Envimet Services power supply system. The laboratory allows to measure the concentrations of:

- SO_2_—continuous automatic measurement using the fluorescence method in accordance with PN-EN 14212: 2013-02/AC: 2014-06E [14];
- NO_x_—continuous automatic measurement using the chemiluminescence method in accordance with PN-EN 14211: 2013-02 [15];
- O_3_—continuous automatic measurement by ultraviolet photometry in accordance with PN-EN 14625: 2013-02 [16];
- PM_10_—continuous automatic measurement with the method for which equivalence with the reference method was demonstrated according to PN-EN 12341: 2014-07 [17];
- PM_2.5_—continuous automatic measurement with the method for which equivalence with the reference method was demonstrated according to PN-EN 12341: 2014-07 [17].

Owing to the cofinancing granted by the Voivodship Fund for Environmental Protection and Water Management in Katowice and by the Silesian University of Technology, the launched mobile laboratory allows to carry out measurements of air pollution concentrations in the vicinity of the selected emissions sources, e.g., energy facilities, municipal sources or sources of fugitive emissions. It is extremely important because it allows to supplement and expand the spectrum of information on air quality in places not covered by systematic monitoring, and to show the significance of the so-called ‘hot spots’.

The article uses the results of measurements made in 2020 on the campus of the Silesian University of Technology: collection point PP: 20B Konarskiego St., 50.292934 N, 18.682164 E.

The town of Gliwice (177,049 inhabitants with a population density of 1322.4 people/km^2^) [18] neighbors on the eastern and southern sides with the towns of Zabrze and Knurów, and on the north and northeastern sides with the poviats of Tarnowskie Góry and Pyskowice, and on the west side with the forest complexes of the poviats of Kędzierzyn-Koźle and Strzelce Opolskie (Opolskie Voivodeship). The sampling point, located in the urban agglomeration with the highest population density in Poland (4,492,300.000 inhabitants of the Śląskie Voivodeship with a population density of 364 people/km^2^) [18], is located on the campus of the Silesian University of Technology. The distance to the nearest building is approx. 12 m and it is greater than the distance required for the sampling point [19]. There are the following communication routes in the vicinity of PP:

- from the northeastern part, approximately 500 m away—road DW902;
- in the northwest direction at a distance of about 450 m—road DW901;
- in the west direction, approximately 600 m away—road DK78.

In addition, in the north direction, at a distance of about 600 m, there is a well-developed railway infrastructure (Gliwice railway station).

In the immediate vicinity of the PP, there is a typical urban development including public utility buildings, a shopping center and multifamily residential buildings. Some of the buildings near the measuring point are connected to the heating system located on the outskirts of the city, approximately 2 km east of the PP.

In addition, there are the following facilities in the vicinity:

- Five steelworks (in the northwest direction (approx. 8.50 km)—Huta Łabędy SA, in the east direction (approx. 8.50 km—Huta Zabrze SA, 13.5 km—Huta Pokój SA, 18.5 km—Huta Batory Ltd.), and in the south direction (at a distance of 20.5 km)—Huta Łaziska S.A.);
- Twenty-nine iron foundries (including GZUT Gliwice (approx. 0.5 km) and Łabędy (approx. 8 km));
- Three coal-fired power plants (in the east direction (approx. 38 km)—power plant Jaworzno, to the south—power plants Łaziska (approximately 21.3 km) and Rybnik (approximately 20.5 km)).

## 3. Results and Discussion

The measurements of PM_10_ (µg/m^3^), PM_2.5_ (µg/m^3^), SO_2_ (µg/m^3^), NO (µg/m^3^), NO_x_ (µg/m^3^) and O_3_ (µg/m^3^) concentrations in the external air for the entire year 2020 concerned only one measuring point (50.292934 N, 18.682164 E), and therefore to define “objects”, an additional parameter (feature) was introduced—time (time moments) of taking the measurements (sampling). Thus, the “objects” described by the above-mentioned features were described in the consecutive weeks of 2020 (due to the limitations of the program MaCzek v. 3.0 (the maximum size of the diagram covers 250 objects and 100 features describing the object), the average values of the concentrations of the above-mentioned parameters were accepted for calculations for the particular weeks). The measurement results were divided into two periods:

- the so-called “winter period”, i.e., from 01/01/2020 to 31/03/2020 and from 01/10/2020 to 31/12/2020;
- the so-called “summer period”, i.e., from 01/04/2020 to 30/09/2020.

The lists of the measured values of PM_10_ (µg/m^3^), PM_2.5_ (µg/m^3^), SO_2_ (µg/m^3^), NO (µg/m^3^), NO_x_ (µg/m^3^) and O_3_ (µg/m^3^) (average daily and weekly values) are presented in Table 1, Table 2 and Table 3.

The atmospheric conditions recorded during the measurement days (wind direction and speed, temperature, air pressure, humidity, etc.) undoubtedly had an impact on the measurement results of the harmful/noxious substance concentrations in the atmosphere. Examples of correlation relationships between the above-mentioned elements are discussed in more detail at the end of the article (Conclusions).

Based on the program MaCzek v. 3.0, the distance types between “objects” (Euclid’s distance) were determined; in the process of variable normalization, data standardization by standard deviation was used. Then, using the measured values of PM_10_ (µg/m^3^), PM_2.5_ (µg/m^3^), SO_2_ (µg/m^3^), NO (µg/m^3^), NOx (µg/m^3^) and O_3_ (µg/m^3^) (VAR1–VAR 6), and the option Simple auto algorithm (the section Order), the diagram was ordered according to an algorithm consisting in selecting objects that are the most similar to each other (Figure 1 and Figure 2).

For both periods (“winter period” and “summer period”), five ranges of similarities (I–V) were defined: from groups that are very similar (distances 0–25.171 for the “winter period” and 0–14.489 for the “summer period”) to very dissimilar groups (distances > 54.439 for the “winter period” and > 40.904 for the “summer period”).

In the case of the “winter period”, five groups were distinguished, and in the case of the “summer period”, seven groups with the greatest similarity (the similarity range I, i.e., very similar groups). In the “winter period”, in this range (Euclid’s distances between objects are not greater than 25.171), the following was distinguished:

- Group no. 1, which includes the weeks numbered: 45 (02/11–08/11), 46 (09/11–15/11) and 51 (14/12–20/12), and, allowing a slightly longer distance, an additional 53rd week (28/12–31/12), which differs to a greater extent from the 51st week (the distance between the 51st and the 53rd week is 29.304). A summary of the basic average measures and measures of relative variability for group no. 1 is presented in Table 4.
- Group no. 2, which includes weeks numbered: 49 (30/11–06/12) and 53 (28/12–31/12), and, allowing a slightly longer distance, an additional 10th week (02/03–08/03), which differs to a greater extent from the week 53rd (the distance between week 53 and week 10 is 26.871). A summary of the basic average measures and measures of relative variability for group no. 2 is presented in Table 5.
- Group no. 3, which includes the weeks numbered: 4 (20/01–26/01), 6 (03/02–09/02) and 9 (24/02–01/03), and, allowing for a slightly longer distance an additional 11th week (09/03–15/03), which differs to a greater extent from the 4th week (the distance between the 11th and 4th week is 33.603). A summary of the basic average measures and measures of relative variability for group no. 3 is presented in Table 6.
- Group no. 4, which includes the weeks numbered: 5 (27/01–02/02), 7 (10/02–16/02), 11 (09/03–15/ 03), 14 (03/03–03/03) and 40 (01/10–04/10). A summary of the basic average measures and measures of relative variability for group no. 4 is presented in Table 7.
- Group no. 5, which includes weeks numbered: 40 (01/10–04/10), 41 (05/10/–11/10), 42 (12/10–18/10), 43 (19/10–25/10), 44 (26/10–01/11), 47 (16/11–22/11), 48 (23/11–29/11) and 52 (21/12–27/12). A summary of the basic average measures and measures of relative variability for group no. 5 is presented in Table 8.

Similarly, (the range of similarity I, i.e., very similar groups), within the “summer period”, we can distinguish the following groups (in this case, the Euclid distances between “objects” do not exceed 14.489):

- group no. 1, which includes weeks no.: 17 (20/04–26/04), 18 (27/04/–03/05) and 19 (04/05–10/05);
- group no. 2, which includes weeks no.: 16 (13/04–19/04), 19 (04/05–10/05), 20 (11/05–17/05) and 21 (18/05 –24/05);
- group no. 3, which includes weeks no.: 22 (25/05–31/05), 23 (01/06–07/06), 24 (08/06–14/06) and 25 (15/06–21/06);
- group no. 4, which includes weeks no.: 25 (15/06–21/06), 29 (13/07–19/07), 32 (03/08–09/08) and 36 (31/08–06/09);
- group no. 5, which includes weeks no.: 30 (20/07–26/07), 31 (27/07–02 08), 34 (17/08–23/08) and 35 (24/08–30/08);
- group no. 6, which includes weeks no.: 27 (29/06–05/07), 28 (06/07–12/07) and 35 (24/08–30/08), or, allowing a slightly longer distance, an additional 26th week (22/06–28/06), which differs more than the other two weeks from the week no. 35;
- group no. 7, which includes weeks no.: 37 (07/09–13/09) and 39 (21/09–27/09), or, allowing a slightly longer distance, an additional 38th week (14/09–20/09), which differs to a greater extent from the 39th week.

An example of a summary of the basic average measures and measures of relative variability for the “summer period” is presented in Table 9.

In the “summer period”, the smallest distances between “objects” were recorded throughout 2020:

- Euclid’s distance between the 30th (20/07–26/07) and the 31st week (27/07–02/08) was 1.694;
- Euclid’s distance between the 30th (20/07–26/07) and the 34th week (17/08–23/08) was 2.336;
- Euclid’s distance between the 31st (27/07–02/08) and the 34th week (17/08–23/08) was 2.717;
- Euclid’s distance between the 27th (29/06–05/07) and the 28th week (06/07–12/07) was 4.230.

Thus, the above “objects”/weeks are the most “similar” to each other due to the simultaneous presence of PM_10_, PM_2.5_, SO_2_, NO, NO_x_ and O_3_ in the atmosphere over the entire period of study time.

The analysis of the results shows that in the weeks of the “summer period” there is a much greater homogeneity of the results (concentrations of the measured substances) as compared to the “winter period”:

- for the group consisting of weeks 26 (22/06–28/06), 27 (29/06–05/07), 28 (06/07–12/07) and 35 (24/08–30/08), the values of the standard deviation of the measurement results of PM_10_ and PM_2.5_ concentrations did not exceed 1.0 µg/m^3^;
- the lowest values of the standard deviation of the SO_2_ concentration (below 1.0 µg/m^3^) were recorded for the groups including, respectively:
- 17th week (20/04–26/04), 18th week (27/04–03/05), 19th week (04/05–10/05) − S (x) = 0.573 µg/m^3^;
- 16th week (13/04–19/04), 19th week (04/05–10/05), 20th week (11/05–17/05), 21st week (18/05–24/05) − S (x) = 0.798 µg/m^3^;
- 30th week (20/07–26/07), 31st week (27/07–02/08), 34th week (17/08–23/08), 35th week (24/08–30/08) − S (x) = 0.604 µg/m^3^;
- 37th week (07/09–13/09), 38th week (14/09–20/09), 39th week (21/09–27/09) − S (x) = 0.686 µg/m^3^;
- the mean values of the coefficients of variation for the groups of weeks in the “summer period” were much below 25%: in the case of PM_10_ and PM_2.5_—12.664%; SO_2_—11.269%; NO—15.816%; NO_x_—9.092%, where the mean value of the coefficient of variation in relation to the content of SO_2_ in the “winter period” was over 32.794% (in the case of the group comprising weeks 27/01–02/02, 10/02–16/02, 09/03–15/03, 30/03–31/03, 01/10–04/10, V (x) exceeded 50%), and for NO, it was more than 20% (21.743%).

## 4. Conclusions

The progress of civilization, apart from the improvement of living standards, also has negative consequences. One of them is manifested as smog, which is a phenomenon effected by the accumulation of a significant amount of pollutants in a given area. The most dangerous effect of smog is the presence of high concentrations of substances hazardous to human life and health in the atmosphere. Exceeding the acceptable standards, especially those of PM_10_, PM_2.5_, nitrogen oxides and benzo(a)pyrene, causes the deterioration of the life quality of the inhabitants, health problems and the growing dissatisfaction of citizens.

Measurements of PM and gas pollutants are carried out not only in air quality monitoring stations (State Environmental Monitoring [20]), but also more and more frequently by mobile measuring points. As part of the article, the authors, using the results of the concentration measurements of PM_10_, PM_2.5_, SO_2_, NO, NO_x_ and O_3_ at the measurement point located on the campus of the Silesian University of Technology (parking lot at Konarskiego 20B; 50.292934 N, 18.682164 E), proposed the use of a symmetrical, square, distance matrix D (Euclid’s distances to determine the similarity/difference of “objects” (in this case, measurement periods, i.e., weeks) and their grouping. The application of Czekanowski’s method allowed for a linear arrangement of the classified “objects” (in the space where dimensions were produced by the measurement results of the concentrations of PM and the above-mentioned chemical compounds) and for the determination of their clusters broken down into the “summer period” (from 01/04/2020 to 30/09/2020) and the “winter period” (from 01/01/2020 to 31/03/2020 and from 01/10/2020 to 31/12/2020). In the “winter period”, there were separated five groups and in the “summer period” there were seven groups with the highest similarity (similarity interval I). For example, in the “winter period”, group no. 1 is formed by weeks (“objects”) no. 45 (02/11–08/11), no. 46 (09/11–15/11), no. 51 (14/12–20/12) and no. 53 (28/12–31/12). In the period from 02/11 to 08/11, the coordinates describing the week were respectively: 39.609 (µg/m^3^) (PM_10_ concentration), 27.726 (µg/m^3^) (PM2.5 concentration), 10.799 (µg/m^3^) (SO_2_ concentration), 20.236 (µg/m^3^) (NO concentration), 53.607 (µg/m^3^) (NO_x_ concentration), 18.328 (µg/m^3^) (O_3_ concentration). Similarly, the coordinates of the other weeks within the group can be determined:

- in the period from 09/11 to 15/11, the descriptive coordinates were, respectively, 49.407 (µg/m^3^) (PM_10_ concentration), 34.585 (µg/m^3^) (PM_2.5_ concentration), 13.512 (µg/m^3^) (SO_2_ concentration), 21.274 (µg/m^3^) (NO concentration), 52.203 (µg/m^3^) (NO_x_ concentration) and 21.683 (µg/m^3^) (O_3_ concentration);
- in the period from 14/12 to 20/12, the descriptive coordinates were, respectively, 53.828 (µg/m^3^) (PM_10_ concentration), 37.680 (µg/m^3^) (PM_2.5_ concentration), 16.560 (µg/m^3^) (SO_2_ concentration), 13.224 (µg/m^3^) (NO concentration), 41.706 (µg/m^3^) (NO_x_ concentration) and 18.528 (µg/m^3^) (O_3_ concentration);
- in the period from 28/12 to 31/12, the descriptive coordinates were, respectively, 41.457 (µg/m^3^) (PM_10_ concentration), 29.020 (µg/m^3^) (PM_2.5_ concentration), 23.863 (µg/m^3^) (SO_2_ concentration), 21.613 (µg/m^3^) (NO concentration), 64.024 (µg/m^3^) (NO_x_ concentration) and 21.507 (µg/m^3^) (O_3_ concentration).

In future, the authors plan to use the above-mentioned method to analyze the results of measurements of air pollution recorded at various points located throughout the entire Silesian agglomeration, which will allow for a better description of the concentration levels of the selected air pollutants in the vicinity of various emissions sources and in places away from already existing monitoring stations. The measurements of PM_10_, PM_2.5_, SO_2_, NO, NO_x_ and O_3_, carried out also on the basis of a mobile laboratory (collecting information about weather conditions in places far away from the existing environmental monitoring stations, or located in the immediate vicinity of kindergartens, schools, hospitals, etc.), will allow, in the opinion of the authors, to obtain more comprehensive knowledge of the risk level posed by substances hazardous to health and will allow to distinguish areas with a similar risk level of the above-mentioned substances. The application of Czekanowski’s model will allow to determine the so-called clusters of objects, taking into account the presence of many features (substances hazardous to human life and health) occurring at the same time. By comparing the measurement results of the concentrations of harmful/onerous substances in the atmosphere and the groupings of “objects” with the observation results of atmospheric conditions (wind direction and speed, temperature, pressure and air humidity, etc.), we can determine the levels of correlation relationships between the above-mentioned elements, which in turn can be used to estimate the risk involving the occurrence of hazards related with air pollution, to develop maps of areas with a similar level of risk and to take preventive action when we have identified “objects” with “similar characteristics”. For example, using the Pearson’s linear correlation coefficient (r_xy_), we can postulate that in the “winter period”, for weeks with the numbers 4 (20/01–26/01), 6 (03/02–09/02), 9 (24/02–01/03) and 11 (09/03–15/03), the correlation relationship (a measure of the strength of a linear relationship) between the concentration levels of PM_10_ and PM_2.5_ and the air temperature is very strongly negative (r_xy_ = −0.937). We also find a negative correlation, but a significant one, between the level of concentrations of PM_10_ or PM_2.5_ and wind speed (r_xy_ = −0.869), the level of PM_10_ or PM_2.5_ content and wind direction (r_xy_ = −0.769) or also of NO_x_ content and wind speed (r_xy_ =−0.744). For comparison, in the case of the weeks numbered 5 (27/01–02/02), 7 (10/02–16/02), 11 (09/03–15/03), 14 (01/ 04–05/04) and 40 (28/09–30/09), the correlation between the level of PM_10_ or PM_2.5_ concentrations and air temperature is only moderately negative (r_xy_ = −0.518). Both in the “winter” and “summer periods”, the correlation dependencies between the content of SO_2_ or NO and air temperature, as well as between air direction and air velocity, are weak (r_xy_ ∈〈0.2; 0.4〉) or they do not show linear relationship (r_xy_ < 0.2). Hence, we can affirm that, in the conditions of the dominant winds from the south and southwest, the main cause of the registered pollutants should be attributed to the emission of pollutants emitted by industrial plants (steelworks Łaziska, power plants Łaziska and Rybnik) and to the heating system based mainly on the use of coal. A consistent implementation of antismog regulations [21], and the use of already existing communication routes located along the longitudinal (DK 78) or close to longitudinal (DW 902) arrangement as a kind of air corridors, especially in the conditions of increasingly stringent emission standards for pollutants produced by internal combustion engines (EURO 6 standard in force since 2014), can be a tool to improve the quality of air pollution in the study area. When formulating conclusions, however, it should be remembered that due to the restrictions introduced by the SARS-CoV-2 virus (from mid-March 2020, the work was performed remotely), the intensity of street traffic, and hence the traffic intensity in the parking lot of the Silesian University of Technology (sampling places) were smaller than during the periods of normal economic functioning and they do not accurately reflect pollution of the atmosphere. According to the General Directorate for National Roads and Motorways, based on obtained data analysis from 32 measuring stations equipped with the viaToll system, the average vehicle traffic from 9 March to 17 May 2020, amounted to approximately 16,500.000 vehicles per day, while in the same period of 2019, it was 25,300.000 vehicles, which means a decrease by about 35%.

## Figures and Tables

**Table 1:** The lists of the measured values of PM_10_ (µg/m^3^), PM_2.5_ (µg/m^3^), SO_2_ (µg/m^3^), NO (µg/m^3^), NO_x_ (µg/m^3^) and O_3_ (µg/m^3^) (average daily).

Data of Measurement	PM_10_ (µg/m^3^)	PM_2.5_ (µg/m^3^)	SO_2_ (µg/m^3^)	NO (µg/m^3^)	NO_x_ (µg/m^3^)	O_3_ (µg/m^3^)
1/01/2020	40.462	28.323	25.478	3.275	13.715	49,103
2/01/2020	124.902	87.432	41.503	38.821	86.583	12,072
3/01/2020	107.254	75.078	32.006	26.421	55.371	25,378
4/01/2020	25.873	18.111	25.046	3.601	13.770	50,112
5/01/2020	23.460	16.422	25.144	4.886	17.680	46,067
6/01/2020	70.488	49.341	33.043	8.248	28.430	23,173
7/01/2020	61.643	43.150	35.222	6.629	29.426	21,879
8/01/2020	54.804	38.363	31.588	8.029	28.292	17,571
9/01/2020	41.634	29.144	27.448	7.653	26.294	26,919
10/01/2020	50.661	35.463	27.321	5.562	19.406	41,867
11/01/2020	30.263	21.184	24.782	6.918	22.376	32,128
12/01/2020	34.582	24.207	27.123	4.682	15.623	46,258
13/01/2020	41.771	29.240	29.442	4.288	22.690	27,935
14/01/2020	45.422	31.795	38.031	26.724	59.833	27,902
15/01/2020	43.022	30.116	13.343	7.803	42.255	29,946
16/01/2020	113.446	79.413	14.942	96.890	183.417	18,787
17/01/2020	174.146	121.902	23.629	112.511	216.371	6929
18/01/2020	42.203	29.542	15.490	5.151	30.294	35,454
19/01/2020	42.173	29.521	6.298	5.523	33.373	22,724
21/01/2020	36.582	25.607	6.424	3.635	25.076	39,900
22/01/2020	34.693	24.285	7.059	5.689	33.262	29,745
23/01/2020	51.923	36.346	6.624	13.620	55.531	26,107
24/01/2020	60.921	42.645	11.049	15.892	49.593	32,822
29/01/2020	18.570	12.999	8.971	2.795	21.635	53,398
30/01/2020	19.767	13.837	7.680	2.671	21.528	48,145
31/01/2020	18.584	13.009	10.025	2.986	22.600	48,406
1/02/2020	14.737	10.316	9.976	5.032	24.268	43,588
2/02/2020	16.204	11.342	6.877	3.420	18.883	60,621
3/02/2020	20.956	14.669	7.283	5.934	31.575	46,431
4/02/2020	19.282	13.497	10.087	7.438	37.422	44,182
5/02/2020	20.682	14.478	7.710	5.456	28.770	49,905
6/02/2020	27.469	19.228	7.229	4.129	30.042	37,740
7/02/2020	55.501	38.851	17.525	28.438	84.398	26,685
8/02/2020	71.518	50.063	18.394	13.235	55.978	26,539
9/02/2020	37.088	25.962	15.105	2.226	24.519	52,930
10/02/2020	19.448	13.614	10.206	2.427	18.100	68,115
11/02/2020	14.693	10.285	8.188	2.099	15.520	68,851
12/02/2020	16.379	11.465	6.434	3.090	18.861	63,740
13/02/2020	35.405	24.783	9.845	9.844	41.556	47,345
14/02/2020	29.596	20.717	9.052	15.384	55.644	36,386
17/02/2020	26.869	18.808	9.978	46.743	96.698	63,471
18/02/2020	19.129	13.390	5.926	35.819	76.429	55,575
19/02/2020	15.076	10.553	6.726	14.019	41.116	52,219
20/02/2020	31.157	21.810	7.897	13.952	48.540	38,597
21/02/2020	22.826	15.978	7.938	8.407	41.231	41,356
22/02/2020	32.567	22.797	9.122	16.796	50.151	46,367
23/02/2020	10.552	7.386	6.357	31.266	66.663	60,459
24/02/2020	16.782	11.747	6.615	8.582	31.868	64,257
25/02/2020	22.354	15.648	7.668	40.552	80.038	40,530
26/02/2020	27.405	19.183	8.930	17.942	50.414	51,332
27/02/2020	45.182	31.627	9.843	12.509	51.201	38,826
28/02/2020	51.275	35.893	13.359	10.519	53.154	30,547
29/02/2020	28.609	20.026	12.959	2.558	23.425	51,959
1/03/2020	26.160	18.312	11.697	3.998	25.008	59,261
2/03/2020	52.902	37.032	13.089	24.511	77.992	28,145
3/03/2020	35.135	24.595	13.523	12.102	56.693	24,492
4/03/2020	48.731	34.112	10.323	29.934	78.973	35,297
5/03/2020	111.741	78.218	16.784	61.991	129.471	33,717
6/03/2020	29.536	20.675	15.393	5.473	35.609	48,829
10/03/2020	39.709	27.796	8.376	13.043	39.571	51,786
11/03/2020	11.191	7.834	6.189	6.445	25.601	56,127
12/03/2020	11.752	8.226	6.141	7.028	24.841	57,496
13/03/2020	18.476	12.933	6.490	5.441	27.422	57,333
14/03/2020	25.766	18.036	7.323	3.747	21.853	65,386
15/03/2020	73.059	51.142	14.093	10.361	36.537	52,678
16/03/2020	35.950	25.165	11.460	7.477	30.866	64,705
17/03/2020	45.950	32.165	10.576	35.557	93.343	45,412
18/03/2020	101.508	71.056	11.884	34.511	86.304	46,020
19/03/2020	69.385	48.570	10.178	31.472	87.208	46,116
20/03/2020	53.433	37.403	13.114	19.665	64.372	33,944
21/03/2020	25.225	17.658	5.552	2.886	15.689	61,926
22/03/2020	23.151	16.206	5.876	1.844	11.355	62,925
23/03/2020	28.758	20.131	8.123	4.722	24.419	61,603
24/03/2020	41.258	28.880	13.065	4.956	28.891	66,584
25/03/2020	40.996	28.697	32.503	4.515	34.613	69,070
26/03/2020	90.548	63.384	27.039	9.403	54.634	52,759
27/03/2020	72.634	50.844	23.487	15.724	66.249	49,757
28/03/2020	87.769	61.438	16.785	8.178	51.397	57,213
29/03/2020	55.676	38.973	7.273	2.754	18.552	65,406
30/03/2020	20.938	14.656	6.421	3.986	21.336	62,598
31/03/2020	39.466	27.626	8.923	5.641	28.951	49,385

**Table 2:** Summary (weekly average values) of the concentration of PM_10_ (µg/m^3^) and PM_2.5_ (µg/m^3^) and the content of SO_2_ (µg/m^3^), NO (µg/m^3^), NO_x_ (µg/m^3^) and O_3_ (µg/m^3^)—“winter period”.

Name	Week	VAR 1	VAR 2	VAR 3	VAR 4	VAR 5	VAR 6
1	01/01–05/01	64.390	45.073	29.835	15.401	37.424	36.546
2	06/01–12/01	49.153	34.407	29.504	6.817	24.264	29.971
3	13/01–19/01	71.740	50.218	20.168	36.984	84.033	24.239
4	20/01–26/01	46.030	32.221	7.789	9.709	40.865	32.143
5	27/01–02/02	17.572	12.301	8.706	3.381	21.783	50.832
6	03/02–09/02	36.071	25.250	11.905	9.551	41.815	40.630
7	10/02–16/02	23.104	16.173	8.745	6.569	29.936	56.887
8	17/02–23/02	22.596	15.817	7.706	23.858	60.118	51.149
9	24/02–01/03	31.109	21.777	10.153	13.809	45.015	48.102
10	02/03–08/03	55.609	38.926	13.823	26.802	75.748	34.096
11	09/03–15/03	29.992	20.995	8.102	7.677	29.304	56.801
12	16/03–22/03	50.657	35.460	9.806	19.059	55.591	51.578
13	23/03–29/03	59.663	41.764	18.325	7.179	39.822	60.342
14 *	30/03–31/03	30.202	21.141	7.672	4.814	25.144	55.992
40 **	01/10–04/10	20.672	14.470	21.566	7.504	27.287	39.777
41	05/10–11/10	24.817	17.372	21.578	11.411	36.492	30.339
42	12/10–18/10	18.573	13.001	21.420	6.437	24.896	31.301
43	19/10–25/10	27.838	19.487	20.970	9.347	32.440	35.988
44	26/10–01/11	25.282	17.698	21.336	10.130	34.463	24.201
45	02/11–08/11	39.609	27.726	10.799	20.236	53.607	18.328
46	09/11–15/11	49.407	34.585	13.512	21.274	52.203	21.683
47	16/11–22/11	32.201	22.541	10.314	8.709	31.882	32.238
48	23/11–29/11	31.438	22.006	10.393	8.332	30.969	23.926
49	30/11–06/12	55.551	38.885	21.415	26.236	65.621	19.920
50	07/12–13/12	71.705	50.193	23.182	20.706	57.192	8.458
51	14/12–20/12	53.828	37.680	16.560	13.224	41.706	18.528
52	21/12–27/12	30.255	21.179	16.119	6.504	24.213	33.171
53	28/12–31/12	41.457	29.020	23.863	21.613	64.024	21.507

Name—no. of week in 2020 r.; VAR 1—concentration of PM_10_ (µg/m^3^); VAR 2—concentration of PM_2.5_ (µg/m^3^); VAR 3—concentration of SO_2_ (µg/m^3^); VAR 4—concentration of NO (µg/m^3^); VAR 5—concentration of NO_x_ (µg/m^3^); VAR 6—concentration of O_3_ (µg/m^3^); *, **—weeks no. 14 and 40 are covered in the list for „winter” and “summer” periods, which results from the adopted beginning/end of the heating and summer seasons.

**Table 3:** Summary (weekly average values) of the concentration of PM_10_ (µg/m^3^), PM_2.5_ (µg/m^3^), SO_2_ (µg/m^3^), NO (µg/m^3^), NO_x_ (µg/m^3^) and O_3_ (µg/m^3^)—“summer period”.

Name	Week	VAR 1	VAR 2	VAR 3	VAR 4	VAR 5	VAR 6
14	01/04–05/04	44.333	31.033	10.803	7.275	30.907	69.658
15	06/04–12/04	54.560	38.192	10.061	16.706	50.848	69.333
16	13/04–19/04	30.405	21.284	8.466	10.930	36.456	69.469
17	20/04–26/04	42.650	29.855	9.710	14.013	45.151	68.260
18	27/04–03/05	38.795	27.157	8.858	16.727	48.588	62.705
19	04/05–10/05	32.864	23.005	8.622	11.708	39.634	65.000
20	11/05–17/05	31.229	21.860	8.627	7.120	30.399	58.528
21	18/05–24/05	24.623	17.236	6.982	7.914	30.229	66.220
22	25/05–31/05	19.375	13.563	6.345	4.566	22.598	50.451
23	01/06–07/06	22.058	15.441	11.419	3.886	22.432	57.825
24	08/06–14/06	28.365	19.856	11.800	4.379	23.697	51.032
25	15/06–21/06	18.806	13.164	11.822	4.887	26.748	46.583
26	22/06–28/06	14.713	10.299	11.433	3.619	19.656	64.836
27	29/06–05/07	14.982	10.487	16.463	4.581	22.476	53.631
28	06/07–12/07	16.113	11.279	18.013	3.350	19.069	52.955
29	13/07–19/07	19.082	13.357	18.005	9.487	32.322	51.518
30	20/07–26/07	21.740	15.218	18.674	6.154	28.075	64.293
31	27/07–02/08	21.681	15.177	18.185	6.274	29.287	63.225
32	03/08–09/08	24.533	17.173	20.187	8.307	33.457	49.824
32	10/08–16/08	week not included in the calculation due to incomplete data					
34	17/08–23/08	20.212	14.148	19.245	6.198	27.547	63.123
35	24/08–30/08	14.243	9.970	19.543	6.323	27.817	55.041
36	31/08–06/09	14.006	9.804	18.882	8.442	29.168	45.852
37	07/09–13/09	25.032	17.552	19.114	20.145	51.944	42.791
38	14/09–20/09	32.529	22.770	19.650	21.463	58.542	39.513
39	21/09–27/09	29.723	20.806	20.475	13.824	46.119	36.864
40	28/09–30/09	23.538	16.477	22.124	13.972	43.441	19.255

**Table 4:** Average measures and measures of relative variability for group no. 1.

Weeks	Parameter	PM_10_	PM_2.5_	SO_2_	NO	NO_x_	O_3_
02/11–08/11, 09/11–15/11, 14/12–20/12, 28/12–31/12	x_min_ (µg/m^3^)	39.609	27.726	10.799	13.224	41.706	18.328
	x_max_ (µg/m^3^)	53.828	37.680	23.863	21.613	64.024	21.683
	$\bar{x}$ (µg/m^3^)	46.075	32.253	16.183	19.087	52.885	20.012
	M(x) (µg/m^3^)	45.432	31.802	15.036	20.755	52.905	20.017
	S(x) (µg/m^3^)	6.692	4.684	5.635	3.952	9.129	1.831
	V(x) (%)	14.524	14.524	34.817	20.706	17.262	9.152

x_min_—minimum value; x_max_—maximum value; $\bar{x}$—arithmetic mean; M(x)—median; S(x)—standard deviation; V(x)—coefficient of variation.

**Table 5:** Average measures and measures of relative variability for group no. 2.

Weeks	Parameter	PM_10_	PM_2.5_	SO_2_	NO	NO_x_	O_3_
30/11–06/12, 28/12–31/12, 02/03–08/03	x_min_ (µg/m^3^)	41.457	29.020	13.823	21.613	64.024	19.920
	x_max_ (µg/m^3^)	55.609	38.926	23.863	26.802	75.748	34.096
	$\bar{x}$ (µg/m^3^)	50.872	35.610	19.700	24.884	68.464	25.174
	M(x) (µg/m^3^)	55.551	38.885	21.415	26.236	65.621	21.507
	S(x) (µg/m^3^)	8.154	5.708	5.235	2.847	6.358	7.767
	V(x) (%)	16.029	16.029	26.574	11.440	9.287	30.854

x_min_—minimum value; x_max_—maximum value; $\bar{x}$—arithmetic mean; M(x)—median; S(x)—standard deviation; V(x)—coefficient of variation.

**Table 6:** Average measures and measures of relative variability for the group no. 3.

Weeks	Parameter	PM_10_	PM_2.5_	SO_2_	NO	NO_x_	O_3_
20/01–26/01, 03/02–09/02, 24/02–01/03, 09/03–15/03	x_min_ (µg/m^3^)	29.992	20.995	7.789	7.677	29.304	32.143
	x_max_ (µg/m^3^)	46.030	32.221	11.905	13.809	45.015	56.801
	$\bar{x}$ (µg/m^3^)	35.801	25.060	9.487	10.186	39.250	44.419
	M(x) (µg/m^3^)	33.590	23.513	9.127	9.630	41.340	44.366
	S(x) (µg/m^3^)	7.313	5.119	1.923	2.585	6.864	10.519
	V(x) (%)	20.428	20.428	20.266	25.377	17.488	23.680

x_min_—minimum value; x_max_—maximum value; $\bar{x}$—arithmetic mean; M(x)—median; S(x)—standard deviation; V(x)—coefficient of variation.

**Table 7:** Average measures and measures of relative variability for group no. 4.

Weeks	Parameter	PM_10_	PM_2.5_	SO_2_	NO	NO_x_	O_3_
27/01–02/02, 10/02–16/02, 09/03–15/03, 03/03–03/03, 01/10–04/10	x_min_ (µg/m^3^)	17.572	12.301	7.672	3.381	21.783	39.777
	x_max_ (µg/m^3^)	30.202	21.141	21.566	7.677	29.936	56.887
	$\bar{x}$ (µg/m^3^)	24.308	17.016	10.958	5.989	26.691	52.058
	M(x) (µg/m^3^)	23.104	16.173	8.706	6.569	27.287	55.992
	S(x) (µg/m^3^)	5.637	3.946	5.947	1.848	3.324	7.308
	V(x) (%)	23.187	23.187	54.266	30.861	12.454	14.038

x_min_—minimum value; x_max_—maximum value; $\bar{x}$—arithmetic mean; M(x)—median; S(x)—standard deviation; V(x)—coefficient of variation.

**Table 8:** Average measures and measures of relative variability for group no. 5.

Weeks	Parameter	PM_10_	PM_2.5_	SO_2_	NO	NO_x_	O_3_
01/10–04/10, 05/10–11/10, 12/10–18/10, 19/10–25/10, 26/10–01/11, 16/11–22/11, 23/11–29/11, 21/12–27/12	x_min_ (µg/m^3^)	18.573	13.001	10.314	6.437	24.213	23.926
	x_max_ (µg/m^3^)	32.201	22.541	21.578	11.411	36.492	39.777
	$\bar{x}$ (µg/m^3^)	26.384	18.469	17.962	8.547	30.330	31.368
	M(x) (µg/m^3^)	26.560	18.592	21.153	8.520	31.425	31.769
	S(x) (µg/m^3^)	4.983	3.488	5.038	1.738	4.449	5.397
	V(x) (%)	18.885	18.885	28.046	20.330	14.667	17.205

x_min_—minimum value; x_max_—maximum value; $\bar{x}$—arithmetic mean; M(x)—median; S(x)—standard deviation; V(x)—coefficient of variation.

**Table 9:** A summary of the basic average measures and measures of relative variability for the “summer period”—examples.

Weeks	Parameter	PM_10_	PM_2.5_	SO_2_	NO	NO_x_	O_3_
20/04–26/04, 27/04/–03/05, 04/05–10/05	x_min_ (µg/m^3^)	32.864	23.005	8.622	11.708	39.634	62.705
	x_max_ (µg/m^3^)	42.650	29.855	9.710	16.727	48.588	68.260
	$\bar{x}$ (µg/m^3^)	38.103	26.672	9.063	14.149	44.457	65.321
	M(x) (µg/m^3^)	38.795	27.157	8.858	14.013	45.151	65.000
	S(x) (µg/m^3^)	4.025	2.817	0.468	2.051	3.688	2.279
	V(x) (%)	10.563	10.563	5.158	14.498	8.296	3.490
13/04–19/04, 04/05–10/05, 11/05–17/05, 18/05 –24/05	x_min_ (µg/m^3^)	24.623	17.236	6.982	7.120	30.229	58.528
	x_max_ (µg/m^3^)	32.864	23.005	8.627	11.708	39.634	69.469
	$\bar{x}$ (µg/m^3^)	29.780	20.846	8.174	9.418	34.180	64.804
	M(x) (µg/m^3^)	30.817	21.572	8.544	9.422	33.428	65.610
	S(x) (µg/m^3^)	3.106	2.175	0.691	1.941	4.026	3.975
	V(x) (%)	10.431	10.431	8.455	20.610	11.779	6.133
07/09–13/09, 21/09–27/09, 14/09–20/09	x_min_ (µg/m^3^)	25.032	17.522	19.114	13.824	46.119	36.864
	x_max_ (µg/m^3^)	32.529	22.770	20.475	21.463	58.542	42.791
	$\bar{x}$ (µg/m^3^)	29.094	20.366	19.746	18.477	52.202	39.723
	M(x) (µg/m^3^)	29.723	20.806	19.650	20.145	51.944	39.513
	S(x) (µg/m^3^)	3.093	2.165	0.560	3.334	5.075	2.424
	V(x) (%)	10.631	10.631	2.837	18.045	9.722	6.102

xmin—minimum value; xmax—maximum value; $\bar{x}$—arithmetic mean; M(x)—median; S(x)—standard deviation; V(x)—coefficient of variation.

## Data Availability

Not applicable.
